# A common base method for analysis of qPCR data and the application of simple blocking in qPCR experiments

**DOI:** 10.1186/s12859-017-1949-5

**Published:** 2017-12-01

**Authors:** Michael T. Ganger, Geoffrey D. Dietz, Sarah J. Ewing

**Affiliations:** 1 Department of Biology, Gannon University, 109 University Square, Erie, PA 16541 USA; 2 Department of Mathematics, Gannon University, 109 University Square, Erie, PA 16541 USA

**Keywords:** Analysis of variance (ANOVA), Blocking, Confidence intervals, Paired and unpaired tests, Statistics, qPCR analysis

## Abstract

**Background:**

qPCR has established itself as the technique of choice for the quantification of gene expression. Procedures for conducting qPCR have received significant attention; however, more rigorous approaches to the statistical analysis of qPCR data are needed.

**Results:**

Here we develop a mathematical model, termed the Common Base Method, for analysis of qPCR data based on threshold cycle values (*C*_*q*_) and efficiencies of reactions (*E*). The Common Base Method keeps all calculations in the logscale as long as possible by working with log_10_(*E*) ∙ *C*_*q*_, which we call the efficiency-weighted *C*_*q*_ value; subsequent statistical analyses are then applied in the logscale. We show how efficiency-weighted *C*_*q*_ values may be analyzed using a simple paired or unpaired experimental design and develop blocking methods to help reduce unexplained variation.

**Conclusions:**

The Common Base Method has several advantages. It allows for the incorporation of well-specific efficiencies and multiple reference genes. The method does not necessitate the pairing of samples that must be performed using traditional analysis methods in order to calculate relative expression ratios. Our method is also simple enough to be implemented in any spreadsheet or statistical software without additional scripts or proprietary components.

## Background

The use of quantitative polymerase chain reaction (qPCR) for diverse applications has increased dramatically [1–4] since its development in the late 1980s [5] and has been established as the technique of choice for the quantification of gene expression [2, 6, 7]. qPCR is a relatively simple technique [8] and amenable to addressing a variety of experimental questions from diverse scientific fields [4]. The protocols and procedures for preparing and processing samples as well as conducting the actual qPCR experiments [4, 7, 9], along with specific concerns and considerations [8, 10–12], have been covered in detail by others. However, data analysis of qPCR is continuing to evolve and the proper use of analysis remains variable in practice (see citations 3–53 in Tellingheusen and Spiess [13] for a comprehensive review).

The output generated by the individual qPCR reactions can be distilled into two values for each well of the qPCR plate: threshold cycle value (*C*_*q*_) and the efficiency of the reaction (*E*). Current methods used to analyze qPCR data utilize at a minimum the *C*_*q*_ values. The *C*_*q*_ values are derived from a logistic curve that plots the growth of a population of amplicons produced through the use of sequence-specific primers [1, 2].

One common method to analyze relative gene expression data is the Livak-Schmittgen [14] method (${2}^{-\Delta \Delta {C}_q}$), which compares two values in the exponent that represent the normalized expression values for a gene of interest in sample type A relative to sample type B.

1 $$ R={2}^{-\left[\left({C}_{q;{GOI}_A}-{C}_{q;{REF}_A}\right)-\left({C}_{q;{GOI}_B}-{C}_{q;{REF}_B}\right)\right]}={2}^{-\left(\Delta  {C}_{q;A}-\Delta  {C}_{q;B}\right)}={2}^{-\Delta  \Delta  {C}_q} $$

Here a gene of interest (*GOI*) in both sample type A and B are normalized using a reference gene (*REF*) and then compared to one another in the exponent. The exponential base of 2 used in this method represents an assumed efficiency of 100% for both genes. This method is simple but ignores the actual efficiency *E* and hence leads to inaccurate results [15, 16].

Since there is no inherent reason to expect the efficiencies for both *GOI* and *REF* to be equivalent or even 100%, most consider it prudent to adjust the expression calculations by incorporating efficiencies into the calculation of relative gene expression [10, 17, 18]. Pfaffl [3] has developed a relative expression ratio (*R*) that incorporates efficiencies into the comparison of *GOI* expression between two sample types.

2 $$ R=\frac{E_{GOI}^{-\left({C}_{q{;}_{GOI_A}}-{C}_{q{;}_{GOI_B}}\right)}}{E_{REF}^{-\left({C}_{q_{;{REF}_A}}-{C}_{q_{;{REF}_B}}\right)}}=\frac{E_{GOI}^{-\Delta  {C}_{q; GOI}}}{E_{REF}^{-\Delta  {C}_{q; REF}}} $$

Schefé et al. [15] show that the calculation and subsequent use of gene-specific efficiencies do alter the relative expression calculations from those derived using the Livak-Schmittgen [14] method. In the Pfaffl [3] method, the difference between the expression of the *GOI* in two sample types is calculated in the exponent of the numerator, while the efficiency of the *GOI* is the exponential base. A similar calculation is done for the *REF* in the denominator. The ratio of the two represents the normalized relative expression of the *GOI* between sample type A and sample type B. In the event that *E* = 2, the two formulas for *R* above coincide. Notice that the efficiencies for the *GOI* (*E*_*GOI*_) and *REF* (*E*_*REF*_) are assumed to be constants across treatments, with efficiencies determined by averaging gene efficiencies across all wells of the qPCR experiment for each gene.

Both methods are widely used and have been generalized to incorporate multiple reference genes [19], as has been recommended for qPCR experiments [11, 20]. Alternatively, Yuan et al. [21] incorporate efficiencies for each gene in each treatment to the overall relative expression calculation through more complex manipulations such as multiple regression and analysis of covariance. The calculations become more complex but do not alter the essentials: *C*_*q*_ comparisons are performed in the exponent of an exponential base that represents the efficiency of the reaction *E*. The equations are constructed to generate a relative expression value by comparing expression in one sample relative to another; a set of relative expression values is then dealt with statistically. In many cases, such a method makes a great deal of sense given the experimental question that is being addressed; however, more complex hypotheses necessitate the ability to perform more complex analyses such as analysis of covariance (ANCOVA) and more elaborate analyses of variance containing more factors and terms that cannot be performed given the existing relative expression equations.

Here we propose the use of individual *E* and *C*_*q*_ values to develop a new Common Base Method and notation that combine the simplicity of the ${2}^{-\Delta \Delta {C}_q}$ method with the greater presumed accuracy of methods including those of Pfaffl [3], Schefé et al. [15], and Yuan et al. [21] that use actual *E* values instead of the theoretical maximum of 2. Specifically, our model uses the experimentally measured efficiency levels *E* of reactions and threshold cycle values *C*_*q*_ but uses a logarithm^1^ to connect them together on the same scale. We examine the numerically equivalent expression ${10}^{\log (E){C}_q}$ and perform our analysis on log(*E*)*C*_*q*_. We also develop logical considerations for the use of unpaired and paired models and suggest the utility of our method for aspects of the general linear model including unpaired and paired *t*-tests and analysis of variance (ANOVA) that otherwise seem less manageable given the non-linear relationship of ${E}^{C_q}$. We show how this approach may be used to analyze the simplest and also most common type of experimental designs where the relative gene expression in one sample type is compared to its expression in another sample type. Finally, a basic spreadsheet or statistical package can be used to implement the Common Base Method to analyze qPCR data for the study of relative gene expression.

## Methods

### The Common Base method

**Fig. 1:**
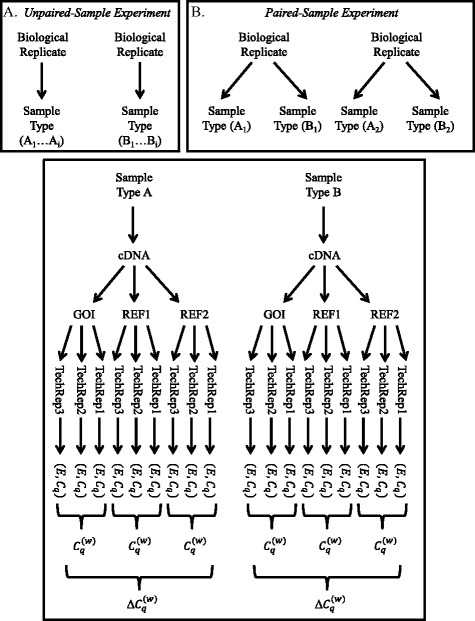
Origin of the Efficiency (*E*) and *C*_*q*_ values. Δ${C}_q^{(w)}$ values are derived from the arithmetic means of the technical replicates. Inset A shows the derivation of sample types A and B in an unpaired sample test where sample types derive from different biological replicates. Inset B shows the derivation of sample types A and B in a paired sample test where sample types derive from the same biological replicate. Please note that each *E* value is logtransformed and multiplied by *C*_*q*_ as discussed in the text. This transformation is not shown in the interest of saving space

Given an experiment or study comparing two populations with biological replicates *r*, sample types *t* [treatment, control, sample type A, sample type B, etc.], genes *g* [gene of interest or reference gene], and technical replicates located in wells *i*, we obtain data points^2^ (*E*, *C*_*q*_) = (*E*_*r*, *t*,  *g*, *i*_, *C*_*q*; *r*, *t*, *g*, *i*_) for each well (Fig. 1).

From each pair of values (*E*,*C*_*q*_), we calculate a single value log_10_(*E*) ∙ *C*_*q*_ , which we call the efficiency-weighted *C*_*q*_ value^3^ (eq. 3). For a fixed biological replicate *r*, sample type *t*, and gene *g*, we then calculate ${C}_q^{(w)}$, the mean efficiency-weighted *C*_*q*_ value over all *n* technical replicate wells, i.e.,

3 $$ {C}_{q;r,t,g}^{(w)}=\frac{1}{n}\sum \limits_{i=1}^n\mathit{\log}\left({E}_{r,t,g,i}\right)\bullet {C}_{q;r,t,g,i} $$

Please note that the superscript (*w*) is a label to denote the use of efficiency-weighting on the *C*_*q*_ values and does not denote exponentiation.^4^ We use the well-specific efficiencies rather than average gene efficiencies. Some have suggested that average gene efficiencies be used [22] because the error in efficiency estimation associated with a single well is likely to be greater than the error in efficiencies between samples amplified with the same primer pair [23]. However, more sophisticated methods of calculating individual well efficiencies are likely to be developed over time that will reduce error in estimation. In any event, the model remains virtually unchanged whether you choose to use well-specific efficiencies or replace them with mean efficiencies. The ultimate choice here is left to the good sense of the researcher.

Given a fixed biological replicate *r*, gene of interest *g* = *GOI*, and a set of *n* reference genes *g* = *REF*_*i*_, we then define the efficiency-weighted Δ*C*_*q*_ value as

4 $$ {\varDelta C}_{q;r,t}^{(w)}={C}_{q;r,t; GOI}^{(w)}-\frac{1}{n}\sum \limits_{i=1}^n{C}_{q;r,t;{REF}_i}^{(w)} $$

which calculates the difference between the weighted ${C}_q^{(w)}$ of the gene of interest and the mean weighted ${C}_q^{(w)}$ of the reference genes (see Table 1 for an illustration of these calculations using a hypothetical data set; Fig. 1). The term $-\frac{1}{n}\sum \limits_{i=1}^n{C}_{q;r,t;{REF}_i}^{(w)}$ of eq. 4 allows for more than one reference gene to be used in the equation. Since our calculations are done in the logscale, we can combine multiple reference genes using the better-known arithmetic mean, whereas other common methods of combining multiple reference genes require the use of geometric means [19]. Computationally, the two methods produce the same results, but we prefer a method that avoids geometric means.

**Table 1:** Sample experimental data from a single qPCR plate for analysis. Hypothetical data are used to show the results of a plate experiment examining the expression of a gene of interest (g) and two reference genes (ref1 and ref2) for two sample types, A and B. The controls for the plate experiment are not shown. $Mean{C}_q^{(w)}$ represents the the arithmetic $Mean{C}_q^{(w)}$ across the three technical replicates

	Well efficiency (*E*)	Gene	*C*_*q*_	log(*E*)	log(*E*) ∙ *C*_*q*_	$Mean{C}_q^{(w)}$
Sample type A	1.844	g	31.246	0.266	8.303	8.397
Sample type A	1.843	g	31.490	0.265	8.360	
Sample type A	1.836	g	32.316	0.264	8.527	
Sample type B	1.839	g	32.565	0.265	8.647	8.611
Sample type B	1.834	g	32.782	0.263	8.631	
Sample type B	1.823	g	32.802	0.261	8.555	
Sample type A	1.905	ref1	26.645	0.280	7.458	7.336
Sample type A	1.886	ref1	26.618	0.276	7.335	
Sample type A	1.868	ref1	26.579	0.271	7.215	
Sample type B	1.918	ref1	26.101	0.283	7.382	7.353
Sample type B	1.906	ref1	26.096	0.280	7.309	
Sample type B	1.915	ref1	26.105	0.282	7.368	
Sample type A	1.900	ref2	26.191	0.279	7.298	7.180
Sample type A	1.881	ref2	25.983	0.274	7.129	
Sample type A	1.879	ref2	25.962	0.274	7.113	
Sample type B	1.890	ref2	25.308	0.277	6.998	7.054
Sample type B	1.883	ref2	25.256	0.275	6.940	
Sample type B	1.911	ref2	25.689	0.281	7.223	
					Sample type A Efficiency-weighted Δ*C*_*q*_	${\Delta C}_{q;r,A}^{(w)}=$1.1387
					Sample type B Efficiency-weighted Δ*C*_*q*_	${\Delta C}_{q;r,B}^{(w)}=$1.4077

The efficiency-weighted ${\Delta C}_q^{(w)}$ values can now be used to calculate a normalized relative expression ratio, but the method of calculation will depend upon whether the experiment uses paired or unpaired data, i.e., whether the biological replicates of sample type A are related to those of sample type B in some paired manner. In terms of calculations and statistical analysis, the difference determines whether a difference of means (unpaired design) or a mean of differences (paired design) is relevant. In either case, we will calculate an efficiency-weighted $\Delta  \Delta  {C}_q^{(w)}$ value as

5 $$ \Delta  \Delta  {C}_q^{(w)}={\varDelta C}_{q;r,A}^{(w)}-{\varDelta C}_{q;r,B}^{(w)} $$

where the terms on the right represent means over all biological replicates of sample type A and sample type B (unpaired design) or corresponding paired samples of types A and B (paired design). In both cases, the relative expression ratio is calculated as

6 $$ R={10}^{-\Delta  \Delta  {C}_q^{(w)}} $$

Given that the ${C}_{q;r,t,g}^{(w)}$ values are calculated from the values log(*E*_*r*, *t*, *g*, *i*_)*C*_*q*; *r*, *t*, *g*, *i*_, and ${10}^{\log (E){C}_q}={10}^{\log \left({E}^{C_q}\right)}={E}^{C_q}$, our calculation of *R* theoretically matches that of Pfaffl [3] and, in the event that *E* reaches the theoretical maximum of 2 (i.e., amplification efficiency is 100%), that of Livak-Schmittgen [14].

Our Common Base Method does not differ in theory from other models, including those of Pfaffl [3], Yuan et al. [21], and Hellemans et al. [19], derived from the Livak-Schmittgen [14] method. Though developed independently, the Common Base Method is computationally similar to eq. 7 of Yuan et al. [21] for relative expression and Tellinghuisen and Speiss [13, 24] (eqs. 7 and 6 respectively) for absolute expression.

## Results

The Common Base Method produces efficiency-weighted $\Delta  {C}_q^{(w)}$ values that may be used to test many different types of hypotheses. Here we show how one may use this method to analyze the simplest type of experiment where one sample type is compared to another. One of the challenges of qPCR, and other plate-based experiments, is that data are derived from qPCR plates that may be run at different times using reagents of differing ages or even using different machines. This challenge results in the potential for large amounts of variation between plates that can obscure trends and make it more difficult to determine differences between treatments. For the following we will consider $\Delta  {C}_q^{(w)}$ values derived from a qPCR plate capable of processing all of the wells of an experiment. We begin with two types of experimental designs: unpaired and paired.

Note that all ensuing data values should be treated as hypothetical values that are provided to illustrate use of the model; the source of the values is thus irrelevant for the following examples. Additionally, we have chosen to present all results in terms of confidence intervals as opposed to standard error. We have made this choice due to the work in the logscale. While we will calculate standard error and confidence intervals in the logscale, we will apply the transformation *y* = 10^*x*^ in the final steps in order to report the relative expression ratio and some form of error bound. While the transformed confidence interval can still be interpreted as a confidence interval placed about the relative expression ratio (although a non-symmetric interval), the transformed standard error cannot be reported as a standard error for the relative expression ratio due to the exponential transformation. Thus, we prefer the simplicity of language that comes from reporting a relative expression ratio and associated confidence interval. We have also arbitrarily chosen 95% confidence levels for the examples, but the actual choice of confidence level is left to the specific researcher dependent upon the norms for a particular experiment.

### Unpaired sample experimental design

For experiments with unpaired samples, the biological replicates of one sample type are not directly linked to replicates of the other sample type. The sample types are derived from distinct biological replicates (Fig. 1, Inset A). Common situations would involve expression of a particular gene between treatment and control or the expression of a particular gene between two genotypes, morphologies, or taxa.

As an example, assume four biological replicates of sample type A and four biological replicates of sample type B from unpaired sources (Table 2). For each replicate *r* from each sample type *t*, we calculate the corresponding ${\Delta C}_{q;r,t}^{(w)}$. Since the replicates are unpaired, we calculate the mean and standard deviation of ${\Delta C}_q^{(w)}$ across the replicates for each sample type. Assuming that relative expression ratio is lognormally distributed, we expect the difference of the mean ${\Delta C}_q^{(w)}$ to follow a normal distribution. To be conservative we assume unequal variances, though this could be tested, between the two sample types and use a two-sample, two-tailed *t*-test (Table 2). The analysis shows an estimated ${\Delta \Delta C}_q^{(w)}$ of 0.7954 – 1.3417 =  − 0.546, a *t*-test statistic of −3.60, 95% confidence interval of (−0.949, −0.143), and *P*-value^5^ of 0.019 using SPSS software [25] and applying the confidence interval formulae of

7 $$ Lower\  CI= mean-{1.96}^{\ast}\frac{SD}{\sqrt{n}}\ \mathrm{and}\  Upper\  CI= mean+{1.96}^{\ast }\ \frac{SD}{\sqrt{n}}. $$

**Table 2:** Results of unpaired *t*-test. An unpaired *t*-test and 95% confidence interval are calculated in SPSS assuming unequal variances using the hypothetical data from Table 1 and three other hypothetical plate experiments. The *P*-value is from a two-tailed test assuming a mean difference of 0

	Sample type A ${\Delta C}_{q;r,A}^{(w)}$	Sample type B ${\Delta C}_{q;r,B}^{(w)}$	
*r* = 1	1.1387	1.4077	
*r* = 2	0.845	1.291	
*r* = 3	0.499	1.496	
*r* = 4	0.699	1.172	
Mean ${\Delta C}_q^{(w)}$	0.7954	1.3417	
SD	0.269	0.141	
N	4	4	
${\Delta \Delta C}_q^{(w)}$			−0.546
T for ${\Delta \Delta C}_q^{(w)}$			−3.60
*P*			0.019
95% CI for ${\Delta \Delta C}_q^{(w)}$			(−0.949, −0.143)
Estimated Expression Ratio ${10}^{-\Delta \Delta {C}_q^{(w)}}$			3.52
95% CI for ${10}^{-\Delta \Delta {C}_q^{(w)}}$			(1.33, 9.29)

The *P*-value shows that ${\Delta \Delta C}_q^{(w)}$ is statistically different from 0 and thus the relative expression ratio is significantly different from 10^−0^ = 1. We estimate that the relative expression ratio is

8 $$ R={10}^{-{\varDelta \varDelta C}_q^{(w)}}={10}^{-\left(-0.546\right)}=3.52 $$

with a 95% *t*-confidence interval of

9 $$ \left({10}^{-\left(-0.124\right)},{10}^{-\left(-0.968\right)}\right)=\left(1.33,9.29\right). $$

In other words, we determine that the gene of interest is expressed at a level 3.52 times higher for members of sample type A compared to members of sample type B (when normalized with respect to the two reference genes) with a 95% confidence level that includes a low of 1.33 and a high of 9.29. We interpret the confidence interval to mean that we are 95% certain that the actual relative expression ratio lies between 1.33 and 9.29. As we have applied an exponential function to the *t*-interval for ${\Delta \Delta C}_q^{(w)}$, this final interval estimate for *R* is not symmetric about 3.52, nor should it be*.* We point out that the confidence interval alternatively can be used to determine the result of the hypothesis test as 1 is not in the interval.

Note that with a qPCR plate with sufficient space for all samples, an analysis of variance (ANOVA) could be used where more than two sample types exist. With a significant ANOVA, post-hoc testing would determine which two groups differ significantly, and corresponding relative expression ratios could be calculated as above since post-hoc testing generally involves applying individual *t*-tests to address comparisons.

Because qPCR experiments are often conducted using multiple plates, variation across qPCR plates is a concern. Such variation can make it more difficult to detect differences in gene expression where such differences exist. One recommendation is to establish each qPCR plate as a complete randomized block [23]. This situation occurs where at least one replicate of each treatment and control is present on a qPCR plate. Blocking factors are often considered as random factors and the interaction between the blocking factor and any main effect is generally not considered [26, 27].

In the following example (Table 3), an experiment is run on two plates, and the plate is the blocking factor for a one-factor ANOVA. The blocking effect’s purpose is to partition variation, and as such the significance of the blocking effect is not relevant to our hypothesis [26]. The results show that we can reject the null hypothesis that all means are the same for the sample types A, B, and C (*P*-value = 0.003). As the means are not all the same, we complete post-hoc *t*-tests for each pair of sample types. After calculating 95% confidence intervals for ${\Delta \Delta C}_q^{(w)}$ and applying the base-10 exponential function, we have 95% interval estimates for the relative expression ratios (1.34, 2.57; Bonferroni-adjusted *P*-value = 0.007) [sample type A vs. B], (0.726, 1.39; Bonferroni-adjusted *P*-value = 1.00) [sample type A vs. C], and (0.391, 0.748; Bonferroni-adjusted *P*-value = 0.007) [sample type B vs. C]. Notice that the first interval exceeds 1, showing that the gene expression for sample type A is significantly larger than that for B. The second interval includes 1, meaning that the gene expression is not significantly different between sample type A and C. The third interval is completely below 1, showing that the gene expression for sample type B is significantly smaller than that for C.

**Table 3:** Analysis of variance (ANOVA) with a blocking factor. Hypothetical data are used to demonstrate an ANOVA for four individuals serving as the replicates spread across two qPCR plates. The qPCR plates serve as a statistical blocking factor. * = expression ratio significantly different from 1

Biological replicate	Group A ${\Delta C}_{q;r,A}^{(w)}$	Group B ${\Delta C}_{q;r,B}^{(w)}$	Group C ${\Delta C}_{q;r,C}^{(w)}$	qPCR plate	Bonferroni-adjusted *P*-value
*r* = 1	0.855	1.203	0.866	1	
*r* = 2	0.711	1.056	0.799	1	
*r* = 3	0.582	0.890	0.522	2	
*r* = 4	0.699	0.775	0.669	2	
Source	Df	MS	F	*P*	
Group	2	0.096	12.864	0.003	
Plate Blocking	1	0.153	20.487	0.002	
Error	8	0.007			
Post-hoc testing	Mean Difference ${\Delta \Delta C}_q^{(w)}$	95% C.I. for ${\Delta \Delta C}_q^{(w)}$	Expression Ratio ${10}^{-\Delta \Delta {C}_q^{(w)}}$	95% C.I. for ${10}^{-\Delta \Delta {C}_q^{(w)}}$	
Group A vs. B	−0.269	(−0.128, −0.410)	1.86	(1.34, 2.57)	0.007*
Group A vs. C	−0.002	(0.139, −0.143)	1.00	(0.726, 1.39)	1.000
Group B vs. C	0.267	(0.408, 0.126)	0.54	(0.391, 0.748)	0.007*

The purpose of blocking is to increase sensitivity by reducing unexplained variation [27]. That is, we are increasing the likelihood of being able to detect significant effects despite the fact that run-to-run variation may be quite large. If the same analysis were performed on data from Table 3, but the blocking factor was not included, then the results would be quite different. Since variation due to the plate-blocking effect is not partitioned, this variation ends up accumulating in the unexplained variation. As such, there would be no effect of treatment on gene expression (F_2,9_ = 4.064; *P*-value = 0.055).

Some [7, 19] have suggested an alternative strategy, the sample maximization method, where separate genes are run on separate qPCR plates. This approach would accomplish the goal of reducing the variation; however, if all samples for an individual gene cannot be run on the same plate, then it would be difficult to partition such variation.

### Paired sample experimental design

For experiments with paired samples, each biological replicate of sample type A is directly paired with a replicate of sample type B. Common situations would involve sample replicates of two types harvested from the same organism or geographic location, or the expression of a particular gene before and after some experimental treatment is applied to an individual (Fig. 1, Inset B). Given a paired experiment we calculate the difference of ${\Delta C}_q^{(w)}$ across the pairs and then calculate the mean of the differences to obtain our $\Delta  \Delta {C}_q^{(w)}$ (as opposed to calculating the mean ${\Delta C}_q^{(w)}$ for each type and then analyzing the difference of means as in the unpaired case; Table 4). Under the assumption of lognormality, we can then apply a two-tailed, paired *t*-test to the data. Similar to the last example^5^, we are testing whether the mean of differences is different from 0.

**Table 4:** Results of paired *t*-test. A paired *t*-test and 95% confidence interval are calculated in SPSS using the hypothetical data from Table 1 and three other hypothetical plate experiments. The *P*-value is from a two-tailed test assuming a mean difference of 0

Biological replicate *r*	Sample A ${\Delta C}_{q;r,A}^{(w)}$	Sample B ${\Delta C}_{q;r,B}^{(w)}$	${\Delta \Delta C}_{q;r}^{(w)}$
*r* = 1	1.1387	1.4077	−0.269
*r* = 2	0.845	1.291	−0.446
*r* = 3	0.499	1.496	−0.997
*r* = 4	0.699	1.172	−0.473
		Mean ${\Delta \Delta C}_q^{(w)}$	−0.546
		SD for ${\Delta \Delta C}_q^{(w)}$	0.314
		N	4
		T for ${\Delta \Delta C}_q^{(w)}$	−3.48
		*P*	0.040
		95% CI for ${\Delta \Delta C}_q^{(w)}$	(−1.046, −0.047)
		Expression Ratio ${10}^{-\Delta \Delta {C}_q^{(w)}}$	3.52
		95% CI for ${10}^{-\Delta \Delta {C}_q^{(w)}}$	(1.11, 11.12)

The analysis shows an estimated mean difference ${\Delta \Delta C}_q^{(w)}$ of −0.546, a *t*-test statistic of −3.48, 95% confidence interval of (−1.046, −0.047), and *P*-value of 0.040 using SPSS software [25]. The *P-*value shows that ${\Delta \Delta C}_q^{(w)}$ is statistically different from 0 and thus the relative expression ratio is significantly different from 10^−0^ = 1. We estimate that the relative expression ratio is

10 $$ R={10}^{-{\varDelta \varDelta C}_q^{(w)}}={10}^{-\left(-0.546\right)}=3.52 $$

with a 95% *t*-confidence interval of

11 $$ \left({10}^{-\left(-0.047\right)},{10}^{-\left(-1.046\right)}\right)=\left(1.11,11.12\right). $$

In other words, we expect that the gene of interest is expressed at a level 3.52 times higher for members of sample type A compared to members of sample type B (when normalized with respect to the two reference genes) with a 95% confidence interval that includes values as low as 1.11 and as high as 11.12. Again, you may note that the interval estimate for *R* is not symmetric about 3.52.

Note that the paired *t*-test utilizes an inherent blocking factor to account for variation among individuals since individuals serve as blocks containing the complete study. The same data in Table 4 could be run as an ANOVA with this blocking factor with no change in *P* value for the main factor.

This paired model may be expanded to include more than two sample types. For example, if gene expression were compared in three organs across several individuals and all of the samples were run on a single qPCR plate, then an ANOVA with a blocking factor would be utilized, where the blocks are individuals (biological replicates) containing each of the three organs. Note, in such a case, gene expression in one type of organ of an individual is likely to be more similar to such organs in other individuals than to other organ types in the same individual. Therefore a blocking factor is appropriate, while a nested model approach would not, though we could conceive of situations where such a nested model would fit.

Given such an experiment we will calculate the difference of ${\Delta C}_q^{(w)}$ across the data within each block (i.e., across each individual) and then perform an ANOVA on the collection of ${\Delta C}_q^{(w)}$ (Table 5). In a standard one-factor ANOVA, the null hypothesis is that the means ${\Delta C}_q^{(w)}$ for each of the three sample types A, B, and C are equal, whereas the alternative hypothesis is that at least one of the means is different from the others.

**Table 5:** Analysis of variance (ANOVA) with a blocking factor. Hypothetical data are used to demonstrate an ANOVA with three groups and four individuals serving as the replicates. The groups in this case are paired within individuals and so the individual serves as a statistical blocking factor. * =  expression ratio significantly different from 1

Biological replicate	Sample type A ${\Delta C}_{q;r,A}^{(w)}$	Sample type B ${\Delta C}_{q;r,B}^{(w)}$	Sample type C ${\Delta C}_{q;r,C}^{(w)}$		Bonferroni-adjusted *P*-value
*r* = 1	0.855	1.408	0.866		
*r* = 2	0.845	1.056	0.799		
*r* = 3	0.499	1.291	0.532		
*r* = 4	0.699	1.172	0.707		
Source	df	MS	F	*P*	
Sample type	2	0.342	20.222	0.002	
Block	3	0.038	2.237	0.184	
Error	6	0.017			
Post-hoc testing	Mean difference ${\Delta \Delta C}_q^{(w)}$	95% C.I. for ${\Delta \Delta C}_q^{(w)}$	Expression ratio ${10}^{-\Delta \Delta {C}_q^{(w)}}$	95% C.I. for ${10}^{-\Delta \Delta {C}_q^{(w)}}$	
Sample type A vs. B	−0.507	(−0.282, −0.732)	3.21	(1.91, 5.40)	0.004*
Sample type A vs. C	−0.002	(0.224, −0.227)	1.00	(0.60, 1.69)	1.000
Sample type B vs. C	0.506	(0.731, 0.281)	0.31	(0.19, 0.52)	0.005*

The analysis shows that we may reject the null hypothesis (*P*-value = 0.002), meaning that at least one of the means is different from the others. We complete post-hoc *t*-tests for each pair of sample types. After calculating 95% confidence intervals for ${\Delta \Delta C}_q^{(w)}$ and applying the base-10 exponential function, we have 95% interval estimates for the relative expression ratios (1.91, 5.40; Bonferroni-adjusted *P*-value = 0.004) [sample type A vs. B], (0.60, 1.69; Bonferroni-adjusted *P*-value = 1.00) [sample type A vs. C], and (0.19, 0.52; Bonferroni-adjusted *P*-value = 0.005) [sample type B vs. C]. Notice that the first interval exceeds 1, showing that the gene expression for sample type A is significantly larger than that for B. The second interval includes 1, meaning that the gene expression is not significantly different between sample type A and C. The third interval is completely below 1, showing that the gene expression for sample type B is significantly smaller than that for C.

More complex blocking would occur where a paired model used more than one qPCR plate. In this case both the individual and the qPCR plate would appear as blocking factors in the statistical model. As discussed previously, our examples above have no nested terms. The interaction terms that include the blocks would not be considered [26]. The exact nature of the model would depend on the design of both the experiment and the qPCR plate setup and warrants a longer exposition.

## Discussion

The advantage of the common base method lies in the use of the common base 10 (or any other base of choice) to force all of the data-based calculations into the logscale and the flexibility to incorporate *E* values into the calculation, however they are derived: sample-specific efficiencies [28], average efficiencies [29], or gene-specific efficiencies [3, 15]. Given experimental evidence that relative gene expression is lognormally distributed [7, 30–32], we expect that $\Delta  \Delta  {C}_q^{(w)}$ approximately follows a normal distribution and can be analyzed using parametric statistical methods (confidence intervals, hypothesis testing, ANOVA, etc.). Without the use of a common base, it is less clear how one should apply these analyses or whether one should do statistics directly on *R* or on log(*R*).

We caution against a few potential pitfalls that may arise from improper analysis of qPCR results. First, avoid grouping data values unless there is a biological motive for the pairing of samples, such as the samples are blocked on the same qPCR plate. For example, the work in Table 4 that calculates ${\Delta \Delta C}_q^{(w)}$ across the table is only valid if the replicates of types A and B are truly paired in some manner and not simply listed next to each other in the table.

Second, use the appropriate type of mean. Averages calculated in the logscale (e.g., ${C}_q^{(w)}$ or ${\Delta C}_{q;r}^{(w)}$) should be done using the standard arithmetic mean (sum the items and divide by *n*), while averages calculated for relative expression ratios should be done with geometric means (multiply the items and take an *n* ^*th*^ root). The different use of means is directly related to the exponential identity *a* ^*x*^ *a* ^*y*^ = *a* ^*x* + *y*^ where addition in the exponent corresponds to multiplication at the base.

Third, ensure that the data used in both the paired and unpaired models conform to the requirements for their use in paired *t*-tests and ANOVAs. The assumptions of such analyses are covered in any general statistics text.

Fourth, apply parametric statistical techniques in the logscale. Evidence suggests that relative expression ratios are lognormally distributed [7, 30–32], and so using parametric statistics on $\Delta  {\Delta C}_q^{(w)}$ appears valid. On the other hand, using parametric statistics directly on relative expression ratios is never valid as the following example shows.

### Example

Consider the paired sample data from Table 4. Suppose that instead of using a paired *t*-test on the $\Delta  {\Delta C}_{q;r}^{(w)}$values, we first calculated the relative expression ratios ${10}^{-\Delta  {\Delta C}_{q;r}^{(w)}}$ for each replicate pair and applied a *t*-test with a hypothesized mean of 1 to those values (Table 6). If we view this experiment as a comparison of A versus B (column 4), then the mean expression ratio is 4.39 and the *P*-value is 0.167, which would be viewed as not significant. We would conclude that expression of the gene in sample types A and B are not significantly different. On the other hand, if we view this experiment as a test of B versus A (column 5), then the mean expression ratio is 0.333 with a *P*-value of 0.005, which shows a significant difference in gene expression. The same data cannot both reject and fail to reject the hypothesis that the relative expression ratio of the sample types is different from 1.

**Table 6:** Results of improper *t*-test usage. An improperly implemented paired *t*-test using hypothetical data from Table 1 and three other hypothetical plate experiments testing the hypothesis of equal gene expression between sample type A and B assuming a mean difference of 0

Biological replicate *r*	Sample A ${\Delta C}_{q;r,A}^{(w)}$	Sample B ${\Delta C}_{q;r,B}^{(w)}$	${10^{-\Delta \Delta C}}_{q;r}^{(w)}$ A vs. B	${10^{\Delta \Delta C}}_{q;r}^{(w)}$ B vs. A
*r* = 1	1.1387	1.4077	1.858	0.538
*r* = 2	0.845	1.291	2.793	0.358
*r* = 3	0.499	1.496	9.931	0.101
*r* = 4	0.699	1.172	2.972	0.337
		Mean	4.39	0.333
		SD	3.73	0.180
		N	4	4
		T	1.82	−7.42
		*P*	0.167	0.005

The interested reader can confirm that our methods are immune to this problem by running a paired *t*-test from the information in Table 4 according to the Common Base Method, but with the A and B columns swapped. This change results in oppositely signed values of ${\Delta \Delta C}_{q;r}^{(w)}$, its mean, the *t*-test statistic, and the confidence interval. The standard deviation and *P*-value remain the same. Consequently, the test will have the same significance result and, after calculating ${10}^{-{\Delta \Delta C}_{q;r}^{(w)}}$, will have the multiplicative inverses of the relative expression ratio and confidence limits.

**Fig. 2:**
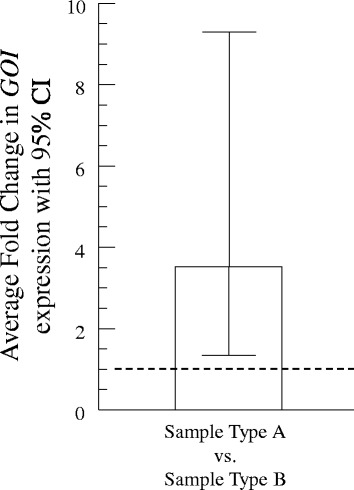
Presentation of results as mean with 95% confidence interval. The results of an unpaired *t*-test using data from Table 2 are graphically shown. The relative expression ratio of the *GOI* is plotted along with the 95% confidence interval

Though analysis is conducted using log-transformed $\Delta  \Delta {C}_q^{(w)}$ values, in most cases it is the relative expression that is of interest. Therefore, we recommend plotting relative expression. We join Yuan et al. [20] in finding the 95% confidence interval to be more meaningful than plotting either standard deviations or standard errors of the mean (Fig. 2) as confidence intervals are more naturally transformed from the logscale to the base level compared to standard deviations or standard errors. The use of confidence intervals is also advocated for other reasons addressed by Colegrave and Ruxton [33], Di Stefano [34], and Nakagawa and Cuthill [35]. Note that for the graphical representation of the ANOVA results with greater than two sample types, the relative expression values would still be plotted. These values would correspond to the post-hoc testing performed.

## Conclusions

In this article we have presented a Common Base Method for use in the statistical analysis of relative expression ratios arising from qPCR experiments. The model is presented in Eqs. 3–6 with examples of its use given in Results.

The Common Base Method has advantages over current methods for analyzing qPCR data. The primary advantage is that the model keeps all calculations in the logscale as long as possible. Staying in the logscale allows one to use arithmetic means instead of geometric means and opens up a larger world of parametric statistical tests that cannot be validly applied at the level of the relative expression ratio. Although we contained our examples to two types of experimental designs, unpaired and paired, the Common Base Method can be adapted to include other analyses within the general linear model. The technique of blocking in such experiments can increase power, and the design of the qPCR plate experiment deserves attention. The use of multiple blocking factors is also possible and the appropriate analysis of such experiments warrants future attention. The potential utility of the Common Base Method suggests that there is great value in determining whether relative expression ratios are lognormally distributed in general. Our method also has the flexibility to be adaptable to efficiency values *E* calculated in a variety of different manners, whether averaged across plates, genes, or from specific wells.

While our use of logscale calculations is not necessarily groundbreaking on its own, we believe that our model presents these concepts in a more accessible manner that will allow easier adaptation for researchers who are not necessarily experts in statistics or bioinformatics. The simplicity of our model and its ability to be quickly calculated in any spreadsheet software is its primary strength.

## Abbreviations

ANOVA: Analysis of variance
CI: Confidence interval
GOI: Gene of interest
qPCR: Quantitative polymerase chain reaction
REF: Reference gene

## Footnotes

1 Although the choice of logarithm is freely made, in our discussion we will always use the base-10 logarithm, denoted by log().

2 The semicolon is used to visually separate the fixed from other data-dependent subscripts.

3 Note that any logarithmic base may be used here as long as the choice is used consistently throughout all future calculations. We recommend the use of either log (base 10) or ln (base *e*) because of their ease of availability in spreadsheet software. On the other hand, using log_2_ (base 2) would allow great analogy with the traditional ΔΔ*C*_*q*_ methods. See footnote 4.

4 To be truly transparent, one should really use a label such as ${C}_q^{\left(w,10\right)}$ to denote weighting with respect to a base 10 logarithm (or more generally, ${C}_q^{\left(w,b\right)}={\log}_b(E)\bullet {C}_q$), but this notation seems overly cumbersome, especially since the choice of base will be made only once and then used consistently throughout all calculations. Notice that if one uses log_2_ and if all efficiencies attain their theoretical maxima of *E* = 2, then log_2_(*E*) ∙ *C*_*q*_ = *C*_*q*_, resulting in the ${2}^{-\Delta \Delta {C}_q}$ model [13]. Thus, our model is a natural generalization of the ${2}^{-\Delta \Delta {C}_q}$ model. Additionally, although different bases *b* produce different values for ${C}_q^{\left(w,b\right)}$, these differences can be ignored for two reasons related to general logarithm properties: (1) ${b}^{C_q^{\left(w,b\right)}}={a}^{C_q^{\left(w,a\right)}}$ for different bases *a* and *b* (thus relative gene expression values will be the same no matter the choice of logarithmic base), and (2) ${C}_q^{\left(w,b\right)}={\log}_{\mathrm{b}}(a)\bullet {C}_q^{\left(w,a\right)}$ and so sums and differences of ${C}_q^{\left(w,b\right)}$ values will be constant multiples of those values calculated via a different base *a*, which means that any parametric statistical tests on differences (*t*-tests, ANOVAs, etc.) will produce results that are statistically the same and are even numerically identical once the functions *b* ^*x*^ or *a* ^*x*^ are applied at the end.

5 We hypothesize that the difference of the means is different from 0 in this example, which corresponds to the relative expression ratio being different from 10^−0^ = 1. If for example we wanted to hypothesize that the relative expression ratio were different by a factor of 2, then we would conduct the *t*-test above with a hypothesized difference of -log(2).

## References

[1] Valasek MA, Repa JJ (2005). The power of real-time PCR. Adv Physiol Educ.

[2] VanGuilder HD, Vrana KE, Freeman WM (2008). Twenty-five years of quantitative PCR for gene expression analysis. BioTechniques.

[3] Pfaffl MW (2001). A new mathematical model for relative quantification in real-time RT-PCR. Nucleic Acids Res.

[4] Taylor S, Wakem M, Dijkman G, Alsarraj M, Nguyen M (2010). A practical approach to RT-qPCR—publishing data that conform to the MIQE guidelines. Methods.

[5] Wang AM, Doyle MV, Mark DF (1989). Quantitation of mRNA by the polymerase chain reaction. Proc Natl Acad Sci.

[6] Ruijter JM, Ramakers C, Hoogaars WMH, Karlen Y, Bakker O, van den Hoff MJB, Moorman AFM (2009). Amplification efficiency: linking baseline and bias in the analysis of quantitative PCR data. Nucleic Acids Res.

[7] Derveaux S, Vandesompele J, Hellemans J (2010). How to do successful gene expression analysis using real-time PCR. Methods.

[8] Radonić A, Thulke S, Mackay IM, Landt O, Siegert W, Nitsche A (2004). Guideline to reference gene selection for quantitative real-time PCR. Biochem Biophys Res Commun.

[9] Bustin SA (2010). Why the need for qPCR publication guidelines?—the case for MIQE. Methods.

[10] Freeman WM, Walker SJ, Vrana KE (1999). Quantitative RT-PCR: pitfalls and potential. BioTechniques.

[11] Bustin SA, Benes V, Garson JA, Hellemans J, Huggett J, Kubista M, Mueller R, Nolan T, Pfaffl MW, Shipley GL, Vandesompele J, Wittwer CQ (2009). The MIQE guidelines: *M*imimum *I*nformation for publication of *Q*uantitative real-time PCR *E*xperiments. Clin Chem.

[12] Bustin SA, Vandesompele J, Pfaffl MW (2009). Standardization of qPCR and RT-qPCR. Genetic Engineering & Biotechnology News.

[13] Tellinghuisen J, Spiess A-N (2014). Comparing real-time quantitative polymerase chain reaction analysis for precision, linearity, and accuracy of estimating amplification efficiency. Anal Biochem.

[14] Livak KJ, Schmittgen TD (2001). Analysis of relative gene expression data using real-time quantitative PCR and the 2^-ΔΔCQ^ method. Methods.

[15] Schefé JH, Lehmann KE, Buschmann IR, Unger T, Funke-Kaiser H (2006). Quantitative real-time RT-PCR data analysis: current concepts and the novel “gene expression’s *CQ* difference” formula. J Mol Med.

[16] Yuan JS, Want D, Stewart CN (2008). Statistical methods for efficiency adjusted real-time PCR quantification. Biotechnol J.

[17] Ramakers C, Ruijter JM, Lekanne Deprez RH, Moorman AFM (2003). Assumption-free analysis of quantitative real-time polymerase chain reaction (PCR) data. Neurosci Lett.

[18] Karlen Y, McNair A, Perseguers S, Mazza C, Mermod N (2007). Statistical significance of quantitative PCR. BMC Bioinformatics.

[19] Hellemans J, Mortier G, De Paepe A, Speleman F, Vandesompele J (2007). qBase relative quantification framework and software for management and automated analysis of real-time quantitative PCR data. Genome Biol.

[20] Udvardi MK, Czechoqski T, Scheible W-R (2008). Eleven golden rules of quantitative RT-PCR. Plant Cell.

[21] Yuan JS, Reed A, Chen F, Stewart CN (2006). Statistical analysis of real-time PCR data. BMC Bioinformatics.

[22] Cook P, Fu C, Hickey M, Han E-S, Miller K. SAS programs for real-time RT-PCR having multiple independent samples. Bioinformatics. 2004;37:990–5. 10.2144/04376BIN02 15597549

[23] Riu I, POwers SJ (2009). Real-time quantitative RT-PCR: design, calculations, and statistics. The Plant Cell.

[24] Tellinghuisen J, Spiess A-N. Statistical uncertainty and its propagation in the analysis of quantitative polymerase chain reaction data: Comparison of methods. Analytical Biochemistry. 2014b;449:94–102. 10.1016/j.ab.2014.06.015 24991688

[25] IBM Corp. Released 2011. IBM SPSS Statistics for Windows, Version 20.0. Armonk, NY: IBM Corp.

[26] Sokal RR, Rohlf FJ (1995). Biometry: the principles and practice of statistics in biological research.

[27] Krzywinski M, Altman N (2014). Analysis of variance and blocking. Nat Methods.

[28] Rao X, Huang X, Zhou Z, Lin X (2013). An improvement of the 2^(−delta delta CT) method for quantitative real-time polymerase chain reaction data analysis. Biostat Bioinforma Biomath..

[29] Ruijter JM, Pfaffl MW, Zhao S, Spiess AN, Boggy G, Blom J, Rutledge RG, Sisti D, Lievens A, De Preter K, Derveaux S, Hellemans J, Vandesompele J (2013). Evaluation of qPCR curve analysis methods for reliable biomarker discovery: bias, resolution, precision, and implications. Methods.

[30] Bengtsson M, Ståhlberg A, Rorsman P, Kubista M (2005). Gene expression profiling in single cells from the pancreatic islets of Langerhans reveals lognormal distribution of mRNA levels. Genome Res.

[31] White AK, VanInsberghe M, Petriv OI, Hamidi M, Sikorski D, Marra MA, Piret J, Aparicio S, Hansen CL (2011). High-throughput microfluidic single-cell RT-qPCR. PNAS.

[32] McDavid A, Finak G, Chattopadyay PK, Dominguez M, Lamoreaux L, Ma SS, Roederer M, Gottardo R (2013). Data exploration, quality control and testing in single-cell qPCR-based gene expression experiments. Bioinformatics.

[33] Colegrave N, Ruxton GD (2002). Confidence intervals are a more useful complement to nonsignificant tests than are power calculations. Behav Ecol.

[34] Di Stefano J (2004). A confidence interval approach to data analysis. For Ecol Manag.

[35] Nakagawa S, Cuthill IC (2007). Effect size, confidence interval and statistical significance: a practical guide for biologists. Biol Rev.

